# Targeted locus amplification to develop robust patient-specific assays for liquid biopsies in pediatric solid tumors

**DOI:** 10.3389/fonc.2023.1124737

**Published:** 2023-04-20

**Authors:** Lieke M. J. van Zogchel, Nathalie S. M. Lak, Nina U. Gelineau, Irina Sergeeva, Ellen Stelloo, Joost Swennenhuis, Harma Feitsma, Max van Min, Erik Splinter, Margit Bleijs, Marian Groot Koerkamp, Willemijn Breunis, Michael Torsten Meister, Waleed Hassan Kholossy, Frank C. P. Holstege, Jan J. Molenaar, Wendy W. J. de Leng, Janine Stutterheim, C. Ellen van der Schoot, Godelieve A. M. Tytgat

**Affiliations:** ^1^ Princess Máxima Center for Pediatric Oncology Research, Utrecht, Netherlands; ^2^ Sanquin Research and Landsteiner Laboratory of the AMC‐ University of Amsterdam, Department of Experimental Immunohematology, Amsterdam, Netherlands; ^3^ Cergentis B.V., Utrecht, Netherlands; ^4^ University Children’s Hospital Zürich, Zürich, Switzerland; ^5^ Oncode Institute, Utrecht, Netherlands; ^6^ Center for Molecular Medicine, University Medical Center (UMC) Utrecht and Utrecht University, Utrecht, Netherlands; ^7^ Department of Pathology, University Medical Center (UMC) Utrecht, Utrecht, Netherlands

**Keywords:** paediatric cancer, neuroblastoma, cell-free DNA (cfDNA), liquid biopsy, patient-specific ddPCR, TLA

## Abstract

**Background:**

Liquid biopsies combine minimally invasive sample collection with sensitive detection of residual disease. Pediatric malignancies harbor tumor-driving copy number alterations or fusion genes, rather than recurrent point mutations. These regions contain tumor-specific DNA breakpoint sequences. We investigated the feasibility to use these breakpoints to design patient-specific markers to detect tumor-derived cell-free DNA (cfDNA) in plasma from patients with pediatric solid tumors.

**Materials and methods:**

Regions of interest (ROI) were identified through standard clinical diagnostic pipelines, using SNP array for CNAs, and FISH or RT-qPCR for fusion genes. Using targeted locus amplification (TLA) on tumor organoids grown from tumor material or targeted locus capture (TLC) on FFPE material, ROI-specific primers and probes were designed, which were used to design droplet digital PCR (ddPCR) assays. cfDNA from patient plasma at diagnosis and during therapy was analyzed.

**Results:**

TLA was performed on material from 2 rhabdomyosarcoma, 1 Ewing sarcoma and 3 neuroblastoma. FFPE-TLC was performed on 8 neuroblastoma tumors. For all patients, at least one patient-specific ddPCR was successfully designed and in all diagnostic plasma samples the patient-specific markers were detected. In the rhabdomyosarcoma and Ewing sarcoma patients, all samples after start of therapy were negative. In neuroblastoma patients, presence of patient-specific markers in cfDNA tracked tumor burden, decreasing during induction therapy, disappearing at complete remission and re-appearing at relapse.

**Conclusion:**

We demonstrate the feasibility to determine tumor-specific breakpoints using TLA/TLC in different pediatric solid tumors and use these for analysis of cfDNA from plasma. Considering the high prevalence of CNAs and fusion genes in pediatric solid tumors, this approach holds great promise and deserves further study in a larger cohort with standardized plasma sampling protocols.

## Introduction

Despite advances in treatment and survival, mortality for pediatric patients with solid tumors that suffer of metastatic or relapsed disease remains high (1–7). During the course of the disease, children face many invasive procedures to acquire tumor material as well as imaging under general anesthesia to determine disease dissemination and response evaluation. Sampling of blood or other liquids produced by the human body, e.g. ‘liquid biopsies’ form a potential source of biomarkers that can be collected in a less invasive manner which could reduce the number of stressful procedures. Moreover, liquid biopsies contain material from both the primary tumor and metastatic lesions, thereby offering a more comprehensive view of the disease and could assist in clinical decision making (8–10). An important challenge is the correct choice of the marker. Studies focusing on the detection of tumor-derived mRNA from blood and bone marrow using a tumor-specific RNA panel have shown promising results for improving risk stratification at diagnosis, as seen in neuroblastoma and rhabdomyosarcoma (10–12). However, these methods still require the use of bone marrow, and the potential for response monitoring with this approach has not been shown yet for rhabdomyosarcoma (10). Cell-free DNA (cfDNA) from plasma holds great potential for diagnostic and prognostic purposes in pediatric solid tumors (13–16). We have previously described hypermethylated *RASSF1A* as a marker for cell free tumor DNA in several pediatric tumors. However, the level of methylation of *RASSF1A* differs in different types of pediatric tumors, which limits its use (16). In contrast to adult malignancies, mutations in pediatric tumors are scarce. Often, they have copy number alterations (CNAs) or translocations resulting in fusion genes which are considered early tumor-driving events and remain present during the entire course of the disease (17–19). In rhabdomyosarcoma, the fusion gene between PAX3 or PAX7 and FOXO1 is an important characteristic within the alveolar subtype (3, 20–22). In Ewing sarcoma, EWSR1 pairs with several fusion partners from the ETS family of transcription factors (5, 17). Neuroblastoma tumors often have amplification of *MYCN*, loss of heterozygosity of chromosome 1p and 11p and gain of 17q (4). Most of these CNAs result in a unique chromosomal fusion, however it is mostly unknown to which chromosome. These genetic events are formed by DNA sequences which are exclusive to a patient, thereby forming a perfect target to detect tumor-derived DNA since these sequences are not present in the background of healthy cell-free DNA, which is always present in blood. For pediatric patients with a solid tumor, fluorescent *in situ* hybridization (FISH), shallow whole genome sequencing (sWGS) or single nucleotide polymorphism (SNP) array has become available for routine diagnostics to identify clinically relevant fusion genes, as well as genomic deletions or amplifications. As these genomic aberrations are independent of gene activity, their presence could potentially be used to detect and quantify tumor burden. Historically, the identification of the exact breakpoint sequence has been time- and resource consuming, as WGS followed by Sanger sequencing validation was necessary (23). However, this procedure can be sped up by using a targeted approach for genomic breakpoint sequencing, like targeted locus amplification/capture (TLA/TLC). TLA/TLC is a technique that uses crosslinking of physically proximal sequences to selectively amplify and sequence regions of >100 kb surrounding specific primer or probe binding sites without prior detailed locus information. TLA can be applied to cells, while TLC is optimized for formalin fixed paraffin embedded (FFPE) material (24–27). The breakpoint sequence revealed by the TLA/TLC technique can be used to design an assay that targets the patient-specific breakpoint. We use droplet digital PCR (ddPCR) to detect these targets in small volumes of plasma from patients with pediatric solid tumors, since ddPCR allows for absolute quantification combined with high sensitivity. In this report, we investigate the possibility of designing patient-specific assays for cell free tumor DNA detection in patients with neuroblastoma, rhabdomyosarcoma and Ewing sarcoma, using TLA/TLC – based breakpoint sequences. Furthermore, we study whether the presence of these specific breakpoints correlates to residual and recurrent disease and, thus, its potential as marker for treatment response.

## Methods

**Figure 1:**
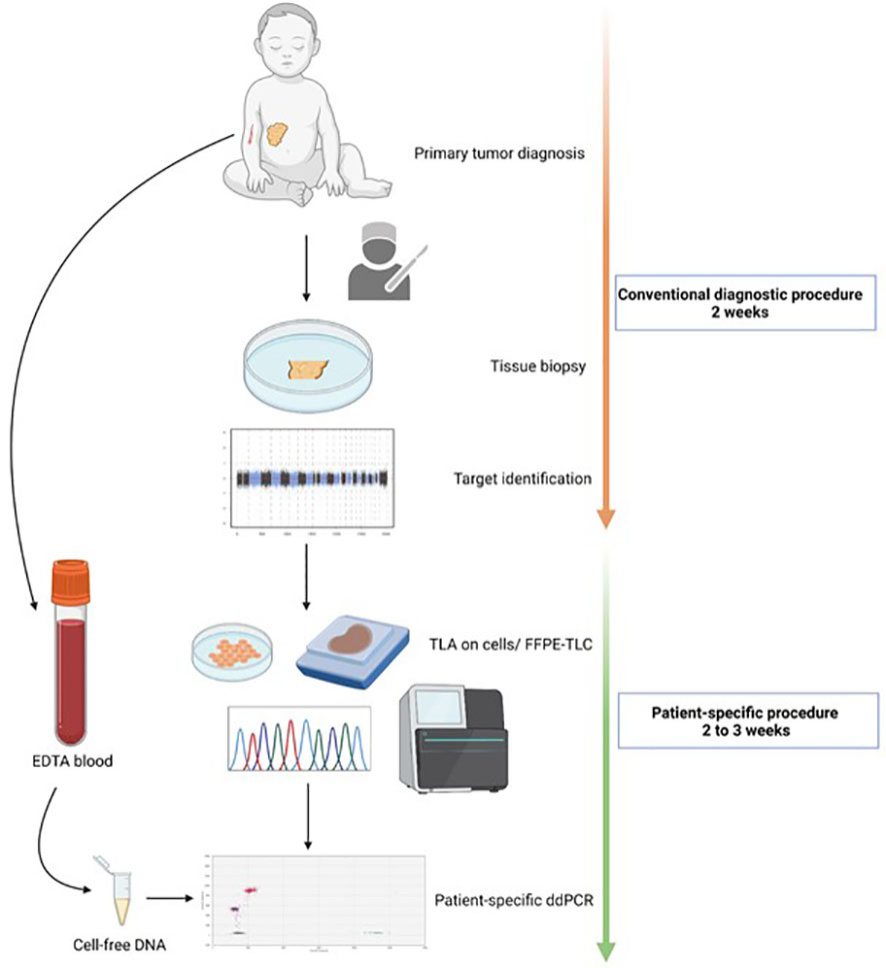
Workflow for the development of a patient-specific assay. At primary diagnosis, tumor material is collected through biopsy or resection. The tissue is then analyzed in the regular diagnostic pipeline. This means copy number analysis through SNP array for neuroblastoma tumors, and fusion gene detection through immunohistochemistry or RT-qPCR for rhabdomyosarcoma and Ewing sarcoma. Based on the identified altered regions/copy number aberrations and fusion partner, for targeted locus amplification (TLA) or targeted locus capture (TLC) is performed on cellsor FFPE material, respectively. The breakpoint sequence(s) are then used for a patient-specific ddPCR design which is then measured on cell- free DNA from EDTA blood.

For a graphical overview of the methods, see **Figure 1**.

### Tumor and plasma samples

Patients with neuroblastoma, rhabdomyosarcoma and Ewing sarcoma, diagnosed in 2016 and 2017 and treated at the Princess Máxima Center (Utrecht, the Netherlands), of whom tumor material (viable or FFPE) and genetic information of the tumor and plasma samples were available, were included in this study. Tumor samples were collected if patients/caretakers gave informed consent for biobanking. Plasma samples from neuroblastoma patients were collected within the Minimal Residual Disease study of the DCOG high-risk protocol (MEC07/219#08.17.0836) and from patients with rhabdomyosarcoma within the Minimal Residual Disease study (add-on within the EpSSG RMS2005, EudraCT number: 2005-000217-35). Plasma samples from the patient with Ewing sarcoma was collected after informed consent for the biobank. Peripheral blood was collected in EDTA tubes (Becton-Dickinson, NJ, USA) and processed within 24 hours. Plasma was obtained by centrifuging the blood samples at 1,375g for 10 minutes and stored at -20°C until further processing.

### Identification of regions of interest

For neuroblastoma tumors, chromosomal regions with aberrations in copy numbers were identified through SNP array. SNP array copy number profiling and analysis of regions of homozygosity were performed according to standard procedures using the CytoSNP-850 K BeadChip (Illumina, San Diego, CA). Visualizations of SNP array results and data analysis were performed using NxClinical software (BioDiscovery, Los Angeles, CA), using Human genome build February 2009 GRCh37/hg1. Chromosomal aberrations that are known to be tumor driving or associated with high- risk disease were preferentially selected for TLA/TLC breakpoint identification (e.g. chromosome 1p, 1q, 2p (including *MYCN* locus), 3p, 11q, 17q) (4). The fusion partners of *FOXO1* in the fusion-positive alveolar rhabdomyosarcomas were validated through RT-qPCR on tumor organoid models (tumoroids) grown from primary tumor material, as described previously (28). In the Ewing sarcoma sample, the fusion between EWSR1 and FLI1 was validated through RT-qPCR on the tumoroid with primers located on EWSR1 exon 8 (AGGAGAGAACCGGAGCATGA) and FLI1 exon 5 (CCCTGAGGTAACTGAGGTGTG).

### Identification of the patient-specific breakpoint(s) using TLA and FFPE-TLC

After the regions of interest (ROI) were identified through standard clinical diagnostic pipelines, ROI-specific primers or probe panels were designed for TLA and FFPE-TLC sequencing by Cergentis (Utrecht, the Netherlands), to sequence tumor-specific breakpoints (24, 25). As starting material for TLA, 2 to 5 million tumoroid cells were used. For tumors for which only FFPE material was available, targeted locus capture (FFPE-TLC) was performed as described previously (27). For FFPE-TLC, 2-3 slides of 10µm with >30% tumor were used. The region-specific primers used for TLA and location of capture probes used for TLC are provided in **Supplemental Table S1**. For TLA, PCR products were library prepped using the Illumina Nextera DNA Flex protocol (Illumina, San Diego, CA, USA) after ROI amplification, whereas for TLC, libraries were created with the KAPA library preparation kit (Roche Kapa Hyperprep, Kapa Unique Dual indexed adapter kit) and subsequently subjected to targeted capture. Sequencing for both TLA and TLC was performed on an Illumina sequencer. 151 bp reads were mapped using BWA-SW, version 0.7.15-r1140, settings bwasw -b 7. The NGS reads were aligned to the human genome (hg19). Breakpoint sites were identified based on coverage peak(s) in the genome and the detection of fusion-reads between different parts of the genome.

### cfDNA isolation and ddPCR

cfDNA was isolated from plasma samples using the Quick-cfDNA Serum & Plasma kit (Zymo Research, CA, USA). Based on the plasma volume available, different amounts of plasma were used to isolate cfDNA based on availability, ranging from 200µl to 1000µl. To correct for variations in the amount of input plasma, cfDNA is reported in copies/mL plasma. In every analysis, *Actin beta* (*ACTB*) was included as a reference gene to determine total cfDNA input.

Using the patient-specific DNA sequence, a ddPCR assay was designed using Primer 3 Plus (https://primer3plus.com/). The design was tested for specificity using Primer Blast (https://www.ncbi.nlm.nih.gov/tools/primer-blast/index.cgi). Designs yielding amplicons in the human reference genome below 1000 bp were excluded to avoid aspecific amplification. The ddPCR assay conditions were optimized using DNA from the primary tumor. In every run, DNA from a healthy leukocyte pool and H_2_O were included as negative controls. The patient-specific primers, probes, and assay conditions are provided in **Supplemental Table S2**.

Reaction mixes for ddPCR were prepared to a final volume of 22 µl using 11 µl ddPCR Supermix for probes (no dUTP) (Bio-Rad Laboratories, Hercules, CA, USA), 1 µl of target assay and 1 µl of *ACTB* assay (final concentration of 900 nM of each primer and 250 nM of each probe, unless otherwise specified), 5 µl of DNA eluate and 3 µl H_2_O. Droplets were generated using the QX200™ Droplet Generator (Bio-Rad) or QX200™ Automated Droplet Generator (Bio-Rad). Incubation and thermal cycling were performed using the C1000 Touch Thermal Cycler (Bio-Rad), with the following program: 95°C for 10 min; 40 cycles of 94°C for 30s, annealing temperature variable per assay, for 1 min; 98°C for 10 min; 4°C hold. Following PCR, droplets were read and quantified using the QX200 Droplet reader and analyzed by QuantaSoft 1.7.4.0917 (Bio Rad) software for single targets on FAM and HEX. Analysis on assays with multiple targets on FAM were done in QX Manager 1.2 Standard Edition software (Bio-Rad). The assay for methylated *RASSF1A* (*RASSF1A*-M) was performed as described previously (16).

## Results

### Patient-specific breakpoints were successfully identified in different pediatric solid tumors

An overview of the clinical characteristics of the patient cohort can be found in **Table 1**. Tumor material grown from primary tumor cells was available for TLA for 2 patients with rhabdomyosarcoma, 1 patient with Ewing sarcoma and 4 with neuroblastoma. For 8 patients with neuroblastoma, FFPE material was available for analysis by FFPE-TLC. An overview of the tested tumor material and identified breakpoints is shown in **Table 2**. In 4 patients with neuroblastoma, multiple breakpoints were detected by TLA and/or FFPE-TLC. In NB2056 2 breakpoints were identified in different locations: between chromosome 2 and 4, and between chromosome 11 and 17. In NB2050 4 breakpoints were identified in chromosome 2. Based on in-silico design results we proceeded with only 2 of these 4 breakpoints for ddPCR design. In 3 samples, NB2066, NB2086 and NB2100, some of the candidate breakpoint sequences were also found in the normal human reference genome (hg38) and therefore were not suited as tumor-specific target. For NB2100, no tumor-specific ddPCR could be designed, in NB2086 and NB2100, other suitable breakpoints were identified in these tumors. In all 14 tumors at least one breakpoint was identified by TLA/FFPE-TLC, and for 13/14 a tumor-specific ddPCR could be designed. These findings illustrate that TLA can be applied successfully both in freshly grown cells and FFPE material, for different tumor entities and different types of genetic aberrations: copy number aberrations and fusion genes.

**Table 1:** Clinical characteristics of patients analyzed by targeted locus amplification (TLA)/capture (TLC).

PtID	Age (years)	Gender	Tumor type	Tumor location	Risk group	Metastatic	Primary tumor volume (ml)	Molecular characteristics	Survival
RMS108	8.8	Female	Fusion-positive rhabdomyosarcoma	Bladder	M	Lung, bone	145.8	*PAX3-FOXO1* (Chr13-Chr2)	2x relapse, died of disease
RMS006	11.6	Male	Fusion-positive rhabdomyosarcoma	Leg	M	Lung, bone	55.3	*PAX3-FOXO1* (Chr13-Chr2)	Progressive disease, died of disease
EWS010			Ewing sarcoma					*EWSR1-FLI* (Chr22-Chr11)	
NB2049	10.8	Male	Neuroblastoma	Abdominal	HR	Bone marrow	3198.8	*MYCN* gain, Chr17 gain	Refractory disease, died of disease
NB2050	4.1	Male	Neuroblastoma	Abdominal	HR	Bone marrow	1276.6	Chr17 gain	Progressive disease, died of disease
NB2053	1.8	Male	Neuroblastoma	Adrenal	HR	Bone marrow, lung, liver	1517	*MYCN* amp, LOH1p, Chr17 gain, *ALK* F1174L mutation	Relapse, died of disease
NB2054	0.7	Male	Neuroblastoma	Adrenal, paravertebral	HR	Bone marrow	2.1	*MYCN* amp, Chr17 gain LOH1p	Complete remission
NB2056	3.2	Female	Neuroblastoma	Adrenal	HR	Bone marrow	210.0	*MYCN* gain, Chr17 gain	Refractory disease, Complete remission
NB2061	0.4	Male	Neuroblastoma	Adrenal	LR	Bone marrow, liver	204	LOH1p	Progressive disease, died of disease
NB2066	1.8	Female	Neuroblastoma	Adrenal	HR	Bone marrow	4.7	*MYCN* gain, Chr17 gain	Complete remission
NB2074	1.9	Male	Neuroblastoma	Adrenal	HR	Bone marrow	491	Chr1p gain, Chr17q gain, *MYCN* amp	Progressive disease, died of disease
NB2086	2.0	Male	Neuroblastoma	Adrenal	HR	Bone marrow	unknown	LOH1p, LOH11q, *MYCN* amp	Progressive disease, died of disease
NB2100	2.4	Male	Neuroblastoma	Adrenal	HR	Bone marrow	1281	LOH1p, gain 17q, *MYCN* amp	Relapse, died of disease
NB2101	4.7	Male	Neuroblastoma	Adrenal	HR	Bone marrow	1767	Chr1p gain, Chr17q gain, *MYCN* amp	Relapse, died of disease

PtID; unique patient identifier; Risk group; M; metastatic, HR; high risk disease; amp, amplification; LOH, loss of heterozygosity.

**Table 2:** Overview of tumor material and breakpoints.

PtID	Tumor material	Timing of tumor sample	Region	Type of genetic aberration	Breakpoint partner 1	Breakpoint partner 2	Details on breakpoint	ddPCR assay successful
**RMS108**	**Tumoroid**	**Relapse surgery**	Chr13-Chr2 (*PAX3-FOXO1*)	Fusion gene	Chr13:41195136 (fwd)	Chr2:223082041	no overlapping base	yes
**RMS006**	**Tumoroid**	**Relapse surgery**	Chr13-Chr2 (*PAX3-FOXO1*)	Fusion gene	Chr13:41136846	Chr2:223082995	1 overlapping base	yes
**ES010**	**Tumoroid**	**Relapse surgery**	Chr22-Chr11 (*EWSR-FLI*)	Fusion gene	Chr22:29292022	Chr11:128772451	2 homologous bases	yes
**NB2049**	**FFPE**	**Primary biopsy**	Chr1-Chr1	Amplification	Chr1:53649791	Chr1:32092640	7 inserted bases	yes
**NB2050** ** ** ** ** ** **	**FFPE** ** ** ** ** ** **	**Resection** ** ** ** ** ** **	Chr2-Chr2	Amplification	Chr2:16022293	Chr2:15960456	5 inserted bases	NA
			Chr2-Chr2	Amplification	Chr2:21514327	Chr2:15957643	4 homologous bases	yes
			Chr2-Chr2	Amplification	Chr2:21511794	Chr2:15957693	2 homologous bases	NA
			Chr2-Chr2	Amplification	Chr2:16108256	Chr2:20989391	3 homologous bases	yes
**NB2053**	**Tumoroid**	**Relapse biopsy**	Chr1-Chr17	Translocation & gain	Chr1:47886678	Chr17:33048245	2 homologous bases	yes
**NB2054**	**FFPE**	**Resection**	Chr2-Chr2	Amplification	Chr2:14863510	Chr2:15987902	1 homologous base	yes
**NB2056** ** **	**FFPE** ** **	**Resection** ** **	Chr4-Chr2	Translocation	Chr4:191044254	Chr2:57488356	1 homologous base	yes
			Chr17-Chr11	Amplification	Chr17:30947919	Chr11:71221924	17 inserted bases	yes
**NB2061**	**Tumoroid**	**Relapse biopsy**	Chr1-Chr16	Translocation	Chr16:68529301	chr1:29295626	2 homologous bases	yes
**NB2066**	**FFPE**	**Primary biopsy**	Chr 3-Chr3	Amplification	Chr3:56630532	Chr3:56630543	20 homologous bases	no, sequence was found in normal hg38 genome
**NB2074**	**FFPE**	**Resection**	Chr2	Amplification	Chr2:18597249	Chr2:27751059		yes
**NB2086** ** **	**Tumoroid** ** **	**Relapse biopsy** ** **	Chr11-9	Translocation	Chr11:88301222	Chr9:92443417		no, sequence was found in normal hg38 genome
			Chr2	Amplification	Chr2:16893201	Chr2:15757504	2 homologous bases	yes
**NB2100**	**Tumoroid ánd FFPE**	**Organoid: relapse biopsy** **FFPE: resection**	Chr1	Deletion	Chr1: 92107327	Chr1: 95347109	1 homologous base	yes
			Chr2	Amplification	Chr2:16670567	Chr2:15943507	2 homologous bases	no, sequence was found in normal hg38 genome
			Chr17	NA			In telomeric sequence	NA
**NB2101**	**FFPE**	**Primary biopsy**	Chr2	Amplification (multiple breakpoints)	Chr2:15179926	Chr2:16075495		yes

PtID, unique patient identifier.

### Results of ddPCR assay in single and multiple breakpoints

**Figure 2:**
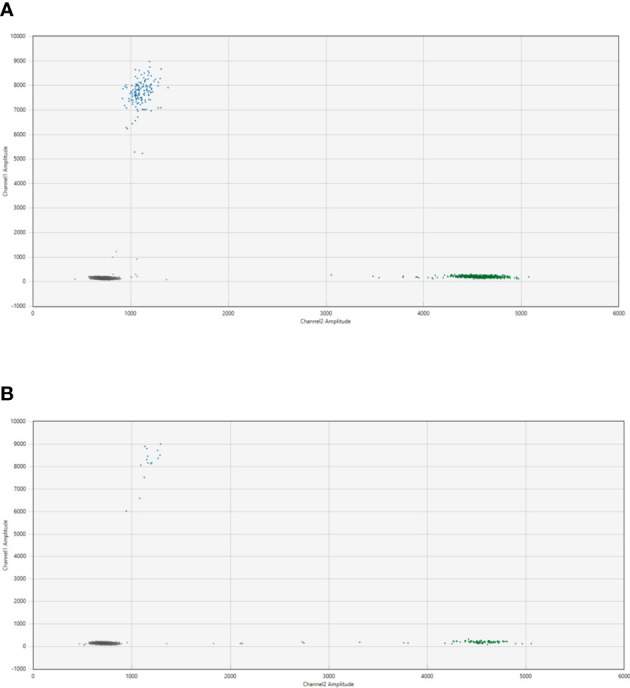
2D plot from the ddPCR assay for NB2049 with **(A)**. cfDNA from the diagnostic plasma sample (total cfDNA input 25.6 ng/well) and **(B)**. positive control with DNA from FFPE material from the primary tumor (total cfDNA input 3.3 ng/well). Blue dots; droplets positive for patient-specific breakpoint (FAM channel) Green dots; droplets positive for *ACTB*(HEX channel) Grey dots; droplets negative for both targets.

**Figure 3:**
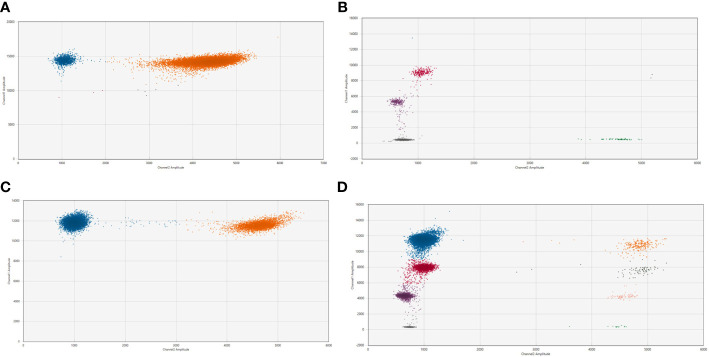
2D plot from the ddPCR assay for NB2050 with 2 patient-specific breakpoints **(A)**. cfDNA from the diagnostic plasma sample (total cfDNA input can not be determined due to overload of the droplets) **(B)**. positive control with DNA from FFPE material from the primary tumor (total cfDNA input 1.7 ng/well). **(C)** Dilution of diagnostic plasma 50 times and **(D)**. 500 times Blue dots; droplets positive for both patient-specific breakpoint (FAM channel) Green dots; droplets positive for *Actin Beta* (HEX channel) Pink dots; droplets positive for breakpoint Chr 2;2 nr 1 Purple dots; droplets positive for breakpoint Chr 2;2 nr 2 (with 450 nM and 125 nM primer and probe concentrations, respectively) Orange dots; droplets positive for both breakpoints and *Actin Beta* Black dots; droplets positive for breakpoint nr 1 and *Actin Beta* Salmon-colored dots; droplets positive for breakpoint nr 2 and *Actin Beta* Grey dot; droplets negative for both targets.

Patient-specific ddPCR assays were designed for 15 breakpoints identified in 13 cases. An illustrative example of a ddPCR assay with one breakpoint is shown in **Figure 2** for the cfDNA from diagnostic plasma and genomic DNA from the primary tumor from patient NB2049. In case more than one tumor-specific ddPCR could be designed, we aimed to combine these in a multiplex assay (**Figure 3**). For NB2050, two breakpoints, both chromosome 2-2 breakpoints in the amplified MYCN locus, were massively amplified relative to the reference gene, resulting in overloading of the droplets and failure to quantify the cfDNA targets accurately in undiluted cfDNA from diagnostic plasma. cfDNA diluted 500 times enabled correct quantification of the different targets. Both **Figures 2**, **3** illustrate the range that can be covered by ddPCR and the possibilities of absolute quantification.

### Presence of patient-specific targets correlates with disease stage

**Figure 4:**
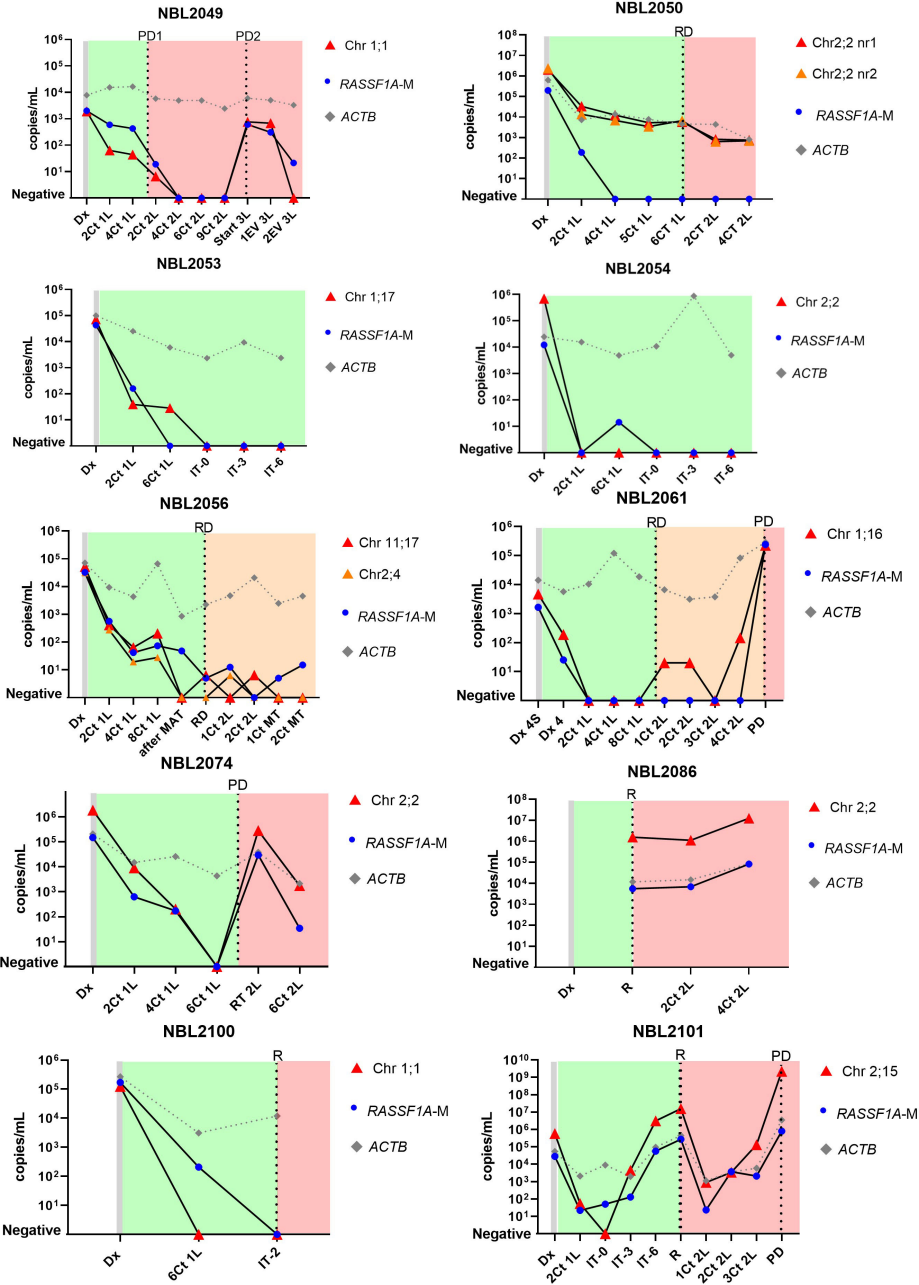
Levels of patient-specific targets, reference gene *ACTIN beta (ACTB)* and methylated *RASSF1A (RASSF1A-M)* in cell-free DNA (cfDNA) from 10 neuroblastoma patients at diagnosis and during the course of the disease. Dx, diagnosis; Dx 4S, diagnosis INSS stage 4S; Dx 4, diagnosis INSS stage 4; nCt 1L, after n courses in first line therapy; nCt 2L, after courses in second line therapy; 3L, third line therapy; 1EV 3L, first evaluation third line therapy; 2EV 3L, second evaluation third line therapy; IT-0, before GD-2 immunotherapy IT-3 after 3 cycles of GD-2 immunotherapy; IT-6, after 6 cycles of GD-2 immunotherapy; MAT, myeloablative therapy and autologous stem cell transplantation; RT 2L, after radiotherapy during second line therapy; MT, maintenance treatment. PD, progressive disease; R, relapse; RD, refractory disease. Green blocks indicate first line treatment, orange blocks indicate added treatment for refractory disease, red blocks indicate treatment for progressive or relapsed disease.

**Figure 5:**
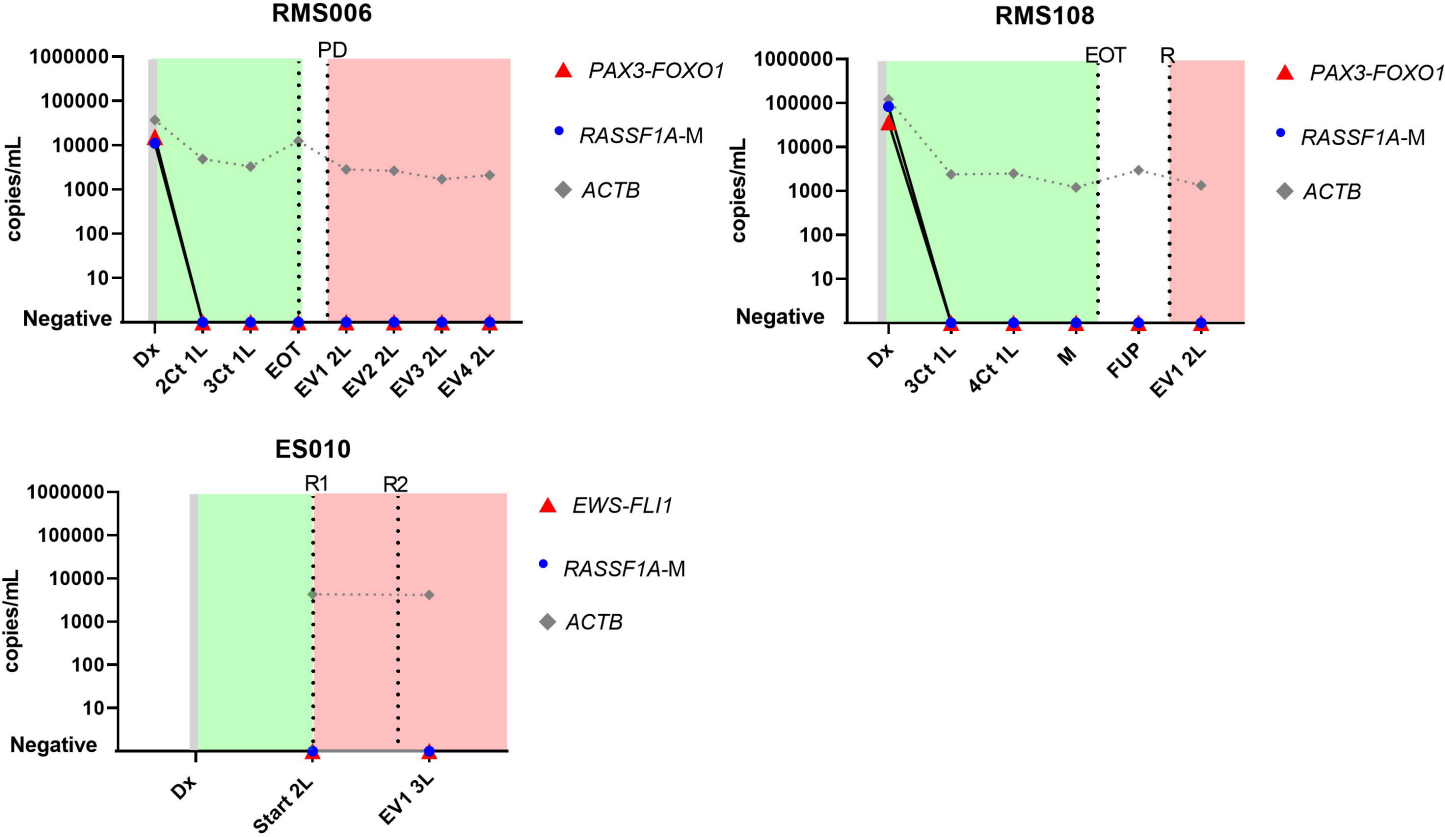
Levels of patient-specific targets, reference gene *ACTIN beta (ACTB)* and methylated *RASSF1A (RASSF1A-M)* in cell-free DNA (cfDNA) in 2 patients with rhabdomyosarcoma (RMS026 and RMS092) and 1 patient with Ewing sarcoma (ES010) at diagnosis and during the course of the disease. Dx, diagnosis; nCt 1L, after n courses in first line therapy; M, maintenance; FUP-follow up; EVn 2L, evaluation number n during second line therapy; EOT, end of treatment; R, relapse; Start 2L, start second line therapy; R, relapse; PD, progressive disease. Green blocks indicate first line treatment, red blocks indicate treatment for progressive or relapsed disease.

Sequential cfDNA samples obtained during the clinical disease course were measured by ddPCR for the patient-specific breakpoint and by the *RASSF1A-*M assay (**Figure 4** for patients with neuroblastoma, **Figure 5** for patients with rhabdomyosarcoma and Ewing sarcoma). In all plasma samples taken at initial diagnosis in patients with neuroblastoma and rhabdomyosarcoma, the patient-specific targets were present. In neuroblastoma, presence of tumor-derived targets followed the clinical course, decreasing after start of treatment and reappearing before or at relapse. In patients NBL2061 and NBL2101 the tumor-specific target is clearly detectable in the cfDNA before a relapse is detected by imaging or standard bone marrow evaluation. In the two patients with rhabdomyosarcoma, the targets in the tumor-derived cfDNA disappeared quickly after start of therapy and did not reappear during therapy for relapse (RMS108) or progressive disease (RMS006). In both cases, no samples were drawn at diagnosis of relapse or progressive disease. For the patient with Ewing sarcoma, the specific breakpoint target was not detected in two cfDNA samples taken during therapy for relapse, even though design of a patient-specific breakpoint was successful, as determined in the positive control. Unfortunately, no sample taken at initial diagnosis was available for this patient.

For NBL2053, NBL2061, NBL2086, NBL2100, RMS006 and RMS108, the tumoroid that was used to identify the patient-specific breakpoint for TLA was grown from a tumor sample taken at relapse. However, we could detect the exact same breakpoints in plasma taken at initial diagnosis. This illustrates clearly that the targeted chromosomal breakpoints in neuroblastoma and the *PAX3-FOXO1* fusion gene in the rhabdomyosarcoma patient remain stable during the course of the disease.

We show that patient-specific targets identified in tumor material by TLA can be detected in cfDNA from diagnostic plasma, furthermore, the presence of these targets track clinical course in neuroblastoma.

### Levels of patient-specific target in cfDNA comparable to RASSF1A-M

Levels of the patient-specific marker and *RASSF1A-*M were comparable at initial diagnosis in patients with neuroblastoma and rhabdomyosarcoma. During the course of treatment minor discrepancies were found between *RASSF1A-M* and the patient-specific marker (NBL2054, NBL2056, NBL2061, NBL2100, NBL2101) (**Figure 4**), reflecting the presence of minimal residual disease. Note that in patient NBL2050 the patient-specific marker targets the highly amplified *MYCN* sequence and therefore has an increased sensitivity compared to the *RASSF1A-M* assay. Similar to the breakpoint levels, all sequential samples in the patients with rhabdomyosarcoma were negative for *RASSF1A-M* (**Figure 5**). For the patient with Ewing sarcoma, all samples were negative for both RASSF1A-M and the breakpoint.

## Discussion

In this study we demonstrate the feasibility to identify a patient-specific target, based on chromosomal structural variants, and design a patient-specific assay for use in liquid biopsies in different pediatric solid tumors. Moreover, we show that the presence of these targets in plasma at initial diagnosis for neuroblastoma and rhabdomyosarcoma, and that its presence during the course of the disease, corresponds to detectable or minimal residual disease status in neuroblastoma.

In patients with neuroblastoma, we observed that the level of tumor-derived cfDNA, as measured by the patient-specific targets, already increased before the clinical diagnosis of relapse or progressive disease was made. This finding suggests a potential for monitoring treatment response in neuroblastoma by detecting tumor-derived cfDNA. This is in line with data from our previous study on hypermethylated *RASSF1A* (16), but is also shown by others. Su et al. reported that the total amount of cfDNA increases before the recurrence of high risk neuroblastoma (29), which can be explained as the majority of the present cfDNA at relapse is tumor derived (16). More recently, Lodrini et al., showed the applicability of detecting tumor-derived cfDNA MYCN and ALK copy number alterations and ALK hotspot mutations in longitudinal plasma samples from patients with neuroblastoma (30). The study of Bosse et al., that predominantly included neuroblastoma patients with an event (91%), showed at least one pathogenic genomic alteration detected in 56% of the samples (31). However, only 20% and 10% of neuroblastoma tumors harbor MYCN amplification or ALK mutation, respectively (4). With the development of TLA/TLC, patient-specific targets for use in liquid biopsies can be detected for any CNAs, as illustrated in our study, which significantly increases the number of patients eligible for monitoring of disease with tumor-derived cfDNA.

In our study, we did not observe re-appearance of the patient-specific breakpoint in samples from patients with rhabdomyosarcoma and Ewing sarcoma. This might be due to a lack of well-timed samples, especially for the patient with Ewing sarcoma. Re-appearance of the patient-specific breakpoint has been described by Eguchi-Ishimae in a patient with fusion-positive rhabdomyosarcoma that suffered from relapse (32). Recently, Ruhen et al. published analysis of cfDNA from plasma in a cohort of 18 patients with rhabdomyosarcoma, which showed a rapid decrease of cfDNA targets after initiation of therapy and an increase at relapse (33). This rapid decrease of tumor-derived cfDNA was also observed by Klega et al. in patients with Ewing sarcoma and fusion-positive rhabdomyosarcoma, often becoming undetectable at the start of the second cycle of chemotherapy (34). Moreover, they observed that in patients with Ewing sarcoma the detection of tumor-derived cfDNA after start of treatment was related to the level of tumor necrosis (34). The relation to tumor burden and monitoring of relapse in Ewing sarcoma was also demonstrated in the recent study by Shulman et al. (35) They also designed a patient-specific assay for the fusion genes in 6 patients with Ewing sarcoma, using data from WGS of the tumor material. In 2 patients that remained in complete remission, the fusion breakpoint disappeared after initiation of treatment. In 4 patients that suffered from relapse, cfDNA levels of the breakpoint reflected presence of relapse and response to therapy (35). These findings from other reports underline the potential of a patient-specific target as a treatment response marker and early relapse detection. But the timing of blood sampling is crucial. In the 2 patients with rhabdomyosarcoma in our study, we did not have samples taken right before or at diagnosis of relapse, only samples taken after start of relapse therapy. Standardized and uniform sampling in a larger cohort of patients with rhabdomyosarcoma and Ewing sarcoma is essential to validate these markers for clinical use. Clinical trials are now being conducted, with liquid biopsy sampling being implemented in the current EpSSG FaR RMS trial for pediatric and adult rhabdomyosarcoma in Europe and in the US a focused trial in adults into liquid biopsies for solid tumors (36, 37).

The use of a patient-specific molecular target as marker for minimal residual disease has been implemented firmly in leukemia (38, 39). In solid tumors, our group has previously described the feasibility to design patient-specific DNA markers from aberrations detected by WGS, and successful marker detection in BM of patients with neuroblastoma (23), but this has not reached clinical practice yet. Some important factors contributing to this lack of translation should be considered. One challenge is defining a target. At initial diagnosis, the search for potential targets can be guided by clinical information and tumor histology, focusing on early oncogenic events that remain present throughout the course of the disease, for example the PAX3-FOXO1 fusion in rhabdomyosarcoma. This fusion gene is considered the tumor-driving event in this tumor entity (3, 20, 40). Also for neuroblastoma, amplification of the MYCN gene, gain of 17q and loss of heterozygosity of chromosome 1 have been found to be recurring events, occurring extremely early in tumor development and remaining present during the course of the disease (41–43). Clonal evolution can affect the suitability of targets. Studies of paired primary and relapse neuroblastoma tumor have shown that mutations detected at relapse represent outgrowth of clones already present at diagnosis or *de novo* events, but most structural events remain present in the relapse sample (41, 44). Combining a panel of targets from diagnosis and then updating this panel again at relapse, using fresh genetic data from the relapsed tumors, might maintain sensitivity of the patient-specific ddPCR assays. In other types of pediatric solid tumors, it might be more challenging to identify patient-specific targets that remain stable throughout the course of the disease. For example, in osteosarcoma, many structural variations have been reported throughout the genome in primary tumors (45, 46), but extensive studies on the stability of these regions in recurrent and progressive disease is lacking. Nonetheless, as many pediatric tumors harbor any structural variant (insertions, deletions, translocations) (18), this approach could benefit cfDNA research in other pediatric tumors as well (42, 45). In our center, regions involved in translocations and copy numbers were previously evaluated by FISH, RT-qPCR and SNP arrays as part of regular clinical investigations and this information can direct the investigations into patient-specific targets. Considering these recurrent regions with copy number aberrations, it would be interesting to explore a multiplex approach of TLA/TLC for neuroblastoma, targeting several regions often carrying amplifications. This approach has shown its potential for detection of translocations in acute leukemia (26).

Another important challenge is the time and effort necessary to identify a patient-specific target. The procedure for FFPE-TLC takes 2 to 3 weeks, shorter than the process based on WGS as described by Subhash et al. (47) Furthermore, FFPE-TLC opens up the possibility to analyze archived samples of patients presenting with late relapse. If no FFPE material is available and not enough cells are available directly after biopsy or surgery, then the time depends on growth of the tumoroids. This can differ significantly. TLA-based approaches to determine the patient-specific breakpoint also preclude the known objections to WGS, with the risk of unsolicited findings and their impact on patients’ lives (48, 49).

In this study, we used a ddPCR-based approach for the detection of the patient-specific targets in cfDNA. Other reports have used hybrid capture sequencing (e.g., TranSS-Seq by Klega et al.) (34). All approaches correlate well with each other (34, 50, 51). The choice of a platform for cfDNA detection depends on the availability at a specific center, the costs and whether multiplexing is necessary, in tumors with multiple targets. A next generation sequencing platform can offer a wider range of targets to be tested, but on the other hand it can be time-consuming to validate, is less flexible and more costly. The possibility of multiplexing targets in cfDNA on the ddPCR is more limited but not impossible, as reported here and in previous publications (52, 53). ddPCR thereby offers a rapid testing modality, also very suited for monitoring of residual disease during treatment and follow-up in a clinical setting.

In some cases, it might be impossible to design a patient-specific assay, which might be due to absence of an appropriate chromosomal region or presence of the sequence in the normal genome (as illustrated by case NB2100). Alternative liquid biopsy-based platforms could be explored, as we have demonstrated previously. We have developed and validated RNA panels for the detection of circulating tumor cells in the cellular compartment of blood and bone marrow for patients with neuroblastoma and rhabdomyosarcoma (10–12). Furthermore, as we also applied in the current study, an enzyme-based ddPCR for methylated RASSF1A in cfDNA is also suited for the detection of tumor-derived cfDNA. Since hypermethylation of RASSF1A has been found in many types of tumors (54–60), this assay offers another approach for liquid biopsy-based disease monitoring. The combination of both RNA and DNA-based platforms for the analysis of liquid biopsies could be complementary, as we have showed previously in a cohort of patients with rhabdomyosarcoma (61).

## Conclusion

In this study, we demonstrate that patient-specific targets can be identified using targeted locus amplification in different pediatric solid tumors. Furthermore, we show that these patient-specific targets can be detected in cfDNA from plasma and their presence may correlate to (minimal) residual or recurrent disease. This approach holds promise for use in daily clinical practice.

## Data availability statement

The original contributions presented in the study are included in the article/**Supplementary Material**. Further inquiries can be directed to the corresponding author.

## Ethics statement

The studies involving human participants were reviewed and approved by medical ethics committees of Academic Medical Center Amsterdam and University Medical Center Utrecht. Written informed consent to participate in this study was provided by the participants’ legal guardian/next of kin.

## Author contributions

LZ, NL, HF, MM, JSt, ESp and GT contributed to conception and design of the study. Acquisition and analysis of results was performed by LZ, NL, NG, IS, ESt, JSw, HF, ESp, MB, MK, WB, MTM, WK, FH, JM and WL. Interpretation of data was done by LZ, NL, IS, ESt, HF, ESp and GT. LZ and NL wrote the first draft of the manuscript. IS, ESt, JSw, HF, ESp wrote sections of the manuscript. All authors contributed to the article and approved the submitted version.
