# Supplementary material for: Targeted locus amplification to develop robust patient-specific assays for liquid biopsies in pediatric solid tumors
Source: Front Oncol. 2023 Apr 20;13:1124737. doi: 10.3389/fonc.2023.1124737 (PMC10157037; doi:10.3389/fonc.2023.1124737)
Supplement: Supplementary file 1 [file Table_1.docx]

| **PtID** | **TLA/TLC** | **Primer name** | **Direction** | **Binding position** | **Sequence** |
| --- | --- | --- | --- | --- | --- |
| **NB2053** | **TLA** | Chr 17 | RV | chr17:33078551 | TCTTTGGGTAACAAGGCTTT |
|  |  |  | FW | chr17:33078702 | AAAGTAAGCATCACTGAGCA |
|  |  | Chr 1 | RV | chr1:47894882 | TCCACATTGCTTGTAAGACA |
|  |  |  | FW | chr1:47894975 | CAGACAAATCCAATGACTGC |
|  |  | Chr 11 | RV | chr11:69560644 | AGTGATTCACAAAGGACACA |
|  |  |  | FW | chr11:69560726 | AGGGACTGGAGCTGATTT |
| **NB2061** | **TLA** | Chr 1 | RV | chr1:29310092 | CAGGCTCAGTAAACAAGGTA |
|  |  |  | FW | chr1:29310232 | AGTATCTGCATCCCTCCAAG |
|  |  | Chr 16-gain | RV | chr16:68569701 | GAATACCGAGAAGCCCAAA |
|  |  |  | FW | chr16:68569926 | CTTACTATTGTGAACTGCGC |
|  |  | Chr 16-gain | RV | chr16:69899652 | TAAGTGTCCATCTCAAAGGG |
|  |  |  | FW | chr16:69899886 | CGACACTGAGGAAAGAAAGA |
|  |  | Chr 16-gain | RV | chr16:71251358 | AGTGTATTTCTACTTGGGCA |
|  |  |  | FW | chr16:71251584 | ATAACTGCTTACTTGTGGGC |
| **NB2086** | **TLA** | Chr 11 | RV | chr11:88295587 | TGCACGGTGAGAATACTTG |
|  |  |  | FW | chr11:88295918 | ACACCTGACACGCCATTT |
|  |  | NMYC | RV | chr2:15785316 | GATCCCTGGTTTCTTTGACT |
|  |  |  | FW | chr2:15785594 | CAATCACGCACCAAATTCC |
|  |  | NMYC | RV | chr2:16886121 | GCTAGAAATGTTCCACCTGT |
|  |  |  | FW | chr2:16886227 | GATATTTAAACCTCAGCTCCTG |
| **NB2100** | **TLA** | Chr 2 | RV | Chr2:15948909 | CTAATTAATTCTCGGCTACACC |
|  |  |  | FW | Chr2:15949614 | TGCTAATTACTTCGCCCTTT |
|  |  | Chr 2 | RV | Chr2:16669425 | TGAATGAATGTGAACAGACAAA |
|  |  |  | FW | Chr2:16669521 | CTTCAGCACATTGGTTGGT |
|  |  | Chr 17 | RV | Chr17:45935137 | CCCACTCCAAGCTACAGG |
|  |  |  | FW | Chr17:45935709 | TAAGCTTGCTTACCTCACTG |
|  |  | Chr 17 | RV | Chr17:45960196 | CTTCACAGTCAGGATTCCAG |
|  |  |  | FW | Chr17:45960293 | AAATGGGCTTGAATGAGTCA |
|  |  | Chr 17 | RV | Chr17:45979674 | GTGTGACCTAACCTCTTTCA |
|  |  |  | FW | Chr17:45979710 | TTACTTTGAGTGGGAGATGG |
|  |  | Chr 17 | RV | Chr17:46000144 | TGGCGAATGTTGACTATTGA |
|  |  |  | FW | Chr17:46000752 | CATAGCTTAAGGGTACGTCC |
|  |  | Chr 1 | RV | Chr17:92088225 | AATGGTCCACTTTGCTCTTT |
|  |  |  | FW | Chr17:92088352 | CCTCTGGCACCCTTGATG |
|  |  | Chr 1 | RV | Chr17:95353980 | CTCAAAGCACATCTGTAGGA |
|  |  |  | FW | Chr17:95354220 | CATCTGACGTCTCACTGAAA |
| **RMS026** | **TLA** | PAX3 exon 9 | RV | chr2:223066247 | ATGACATTGTCAGCCTGTAG |
|  |  |  | FW | chr2:223066337 | CATATGATCCTGGAGCTGAC |
|  |  | PAX3 exon 7 | RV | chr2:223086048 | TGGCTTTCAACCATCTCATT |
|  |  |  | FW | chr2:223086281 | GTGTCAAAGGTCAGTAGAGG |
| **RMS092** | **TLA** | PAX3 exon 9 | RV | chr2:223066247 | ATGACATTGTCAGCCTGTAG |
|  |  |  | FW | chr2:223066337 | CATATGATCCTGGAGCTGAC |
|  |  | PAX3 exon 7 | RV | chr2:223086048 | TGGCTTTCAACCATCTCATT |
|  |  |  | FW | chr2:223086281 | GTGTCAAAGGTCAGTAGAGG |
| **ES010** | **TLA** | EWSR1 | RV | chr22:29,287,610 | CATCCAAGATGTTAGCTGGA |
|  |  |  | FW | chr22:29,287,807 | CTATTGCAGGCCACTATGAT |
|  |  | FLI1 | RV | chr11:128,786,438 | ATGTACGAACGTACAGTTGT |
|  |  |  | FW | chr11:128,786,734 | CAATCAGCACATCTCTTCCT |
|  |  |  |  |  |  |
| **PtID** | **TLA/TLC** | **Probe targeted region (hg19)** | | | |
| **NB2049** | **TLC** | chr1:53625000-53665000 | | | |
| **NB2050** | **TLC** | chr2:15952000-15962000 | | | |
|  |  | chr2:16104000-16114000 | | | |
| **NB2054** | **TLC** |  | | | |
| **NB2056** | **TLC** | chr2:57465000-57515000 | | | |
|  | **TLC** | chr11:71200000-71250000 | | | |
| **NB2066** | **TLC** | chr3:56610000-56650000 | | | |
| **NB2074** | **TLC** | chr2:18585000-18615000 | | | |
|  |  | chr2:27740000-27780000 | | | |
|  |  | chr2:31100000-31130000 | | | |
| **NB2100** | **TLC** | chr2:15928000-15958000 | | | |
|  |  | chr2:16655000-16685000 | | | |
| **NB2101** | **TLC** | NA, SV identified with sWGS FFPE-TLC preps | | | |

**Supplemental Table S1. Primer location and sequences for TLA, and capture probe locations for TLC**

PtID= unique patient identifier
