# Supplementary material for: Targeted locus amplification to develop robust patient-specific assays for liquid biopsies in pediatric solid tumors
Source: Front Oncol. 2023 Apr 20;13:1124737. doi: 10.3389/fonc.2023.1124737 (PMC10157037; doi:10.3389/fonc.2023.1124737)
Supplement: Supplementary file 2 [file Table_2.docx]

| **PtID** | **Region** | **Start** | **End** | **Oligo** | **Oligo sequence (5'-3')** | **Amplicon size (bp)** | **Reporter** | **Quencher** | **Annealing temperature (°C)** |
| --- | --- | --- | --- | --- | --- | --- | --- | --- | --- |
| **NB2049** | Chr1-1 | 53625000 | 53665000 | Fwd | ATAACCTGGTTCATGCCATC | 111 | FAM | ZEN/Iowa Black™ FQ | 57 |
|  |  |  |  | Rev | CAGAGTCACACAGGCAGAAA |  |  |  |  |
|  |  |  |  | Probe | AGCTGGATGTGGTGAAAGGCT |  |  |  |  |
| **NB2050** | Chr2-chr2 | 15952000 | 15962000 | Fwd | CTCCTGTCTACCAGGAAGTG | 100 | FAM | ZEN/Iowa Black™ FQ | 57 |
|  |  |  |  | Rev | TGCTTGGTTCTATGACGAGA |  |  |  |  |
|  |  |  |  | Probe | ACTCTACTTCCAGGAGATCTTTTTGTAGA |  |  |  |  |
|  | Chr2-Chr2 | 15952000 | 15962000 | Fwd² | CCTTATACCCTGGCCTTCC² | 117 | FAM | ZEN/Iowa Black™ FQ | 57 |
|  |  |  |  | Rev² | ACAGACAGGGGTTGGGAAC² |  |  |  |  |
|  |  |  |  | Probe¹'³ | TGCCTGCACATAGGCCCAT³ |  |  |  |  |
| **NB2053** | Chr1-Chr17 | 47886678 | 33048245 | Fwd | CCATCAGTCCAGATGAGCAG | 94 | FAM | ZEN/Iowa Black™ FQ | 59 |
|  |  |  |  | Rev | TGTAACTATGCAGCCCTGTG |  |  |  |  |
|  |  |  |  | Probe¹ | TGGGGCATCTCTCCCAGAACCCTCCA |  |  |  |  |
| **NB2054** | Chr2-Chr2 | 14863510 | 15987902 | Fwd | ACCATGGAAACCATGAGACA | 123 | FAM | ZEN/Iowa Black™ FQ | 59 |
|  |  |  |  | Rev | ATTACAGGTGCCTACCACAC |  |  |  |  |
|  |  |  |  | Probe¹ | ACTGTCAGTTTCACTCATTTCCGCAGCACA |  |  |  |  |
| **NB2056** | Chr4-chr2 | 57465000 | 57515000 | Fwd | GGGTTAGGGTTCGGGTTT | 116 | FAM | ZEN/Iowa Black™ FQ | 57 |
|  |  |  |  | Rev | CAAAATGCAGGGATTACAGG |  |  |  |  |
|  |  |  |  | Probe | AAAACGGAGACCAGGAGCG |  |  |  |  |
|  | Chr 17/11 | 71200000 | 71250000 | Fwd² | GCACTTTGGATAAGGTATACTCAA | 119 | FAM | ZEN/Iowa Black™ FQ | 57 |
|  |  |  |  | Rev² | GTCCCTGTTCCTTCCCCTA |  |  |  |  |
|  |  |  |  | Probe³ | TGTATATATGGTTCATGGATACGACC |  |  |  |  |
| **NB2061** | Chr16-Chr1 | 68529301 | 29295626 | Fwd | CAGAGTTTCACTCTTGCTGC | 81 | FAM | ZEN/Iowa Black™ FQ | 59 |
|  |  |  |  | Rev | CTTGGGTGACAGGGCAAG |  |  |  |  |
|  |  |  |  | Probe | AGATCATGCCATTGCACTCCAGCCTGG |  |  |  |  |
| **NB2074** | Chr2-Chr2 amplification | 31120077 | 98796284 | Fwd | GCCTGCCCTTTCTTGTTTC | 106 | FAM | ZEN/Iowa Black™ FQ | 57 |
|  |  |  |  | Rev | GAGGGAGGAAGGAGAGAGAA |  |  |  |  |
|  |  |  |  | Probe¹ | ACAAGCACAGGCTGAAGACAAGCACA |  |  |  |  |
| **NB2086** | Chr2-Chr2 amplification | 16893201 | 15757504 | Fwd | AACAAAGGATATTACCCATCT | 120 | FAM | MGB Eclipse® | 55 |
|  |  |  |  | Rev | AGGTAGTAGGATCATGACTGAA |  |  |  |  |
|  |  |  |  | Probe¹ | TGCCATTGTAGTATGGA |  |  |  |  |
| **NB2100** | Chr 1-Chr1 deletion | 92107327 | 95347109 | Fwd | CTCTTTTTCAGCCAGGCGT | 75 | FAM | ZEN/Iowa Black™ FQ | 55 |
|  |  |  |  | Rev | GCTGGGACTACAGGCACC |  |  |  |  |
|  |  |  |  | Probe | AATTTTAGCCAGGCATGGTGGCG |  |  |  |  |
| **NB2101** | Chr2-Chr2 amplification | 15179926 | 16075495 | Fwd | CACCTTTAGCAGAGCTTGGA | 118 | FAM | ZEN/Iowa Black™ FQ | 57 |
|  |  |  |  | Rev | GACAATCAGTCAGGTGGAGG |  |  |  |  |
|  |  |  |  | Probe¹ | AGGACAGCCTGGGAGGCTGATCATCTCC |  |  |  |  |
| **RMS026** | Chr13-Chr2 (*PAX3-FOXO1*) | 41136846 | 223082995 | Fwd | GTAGACATGGGGTTTCACC | 141 | FAM | ZEN/Iowa Black™ FQ | 58 |
|  |  |  |  | Rev | TCCTGGTCTAGGATCTTGTC |  |  |  |  |
|  |  |  |  | Probe | TGACTAAAACCTCCTGCATCTGTTT |  |  |  |  |
| **RMS092** | Chr13-Chr2 (*PAX3-FOXO1*) | 41136846 | 223082995 | Fwd | AAGTAGAATTGCTAGAATGTG | 116 | FAM | ZEN/Iowa Black™ FQ | 55 |
|  |  |  |  | Rev | AGTCCTGCTTCTCTATTCCT |  |  |  |  |
|  |  |  |  | Probe | TGCAGTTGTGGGTTTGTATCTGT |  |  |  |  |
| **ES010** | Chr 22-Chr11 (*EWS-FLI*) | 29292022 | 128772451 | Fwd | GCTCCATTTTAGCAGTGCG | 111 | FAM | ZEN/Iowa Black™ FQ | 58 |
|  |  |  |  | Rev | GGAGAGCAGTTGGAACCTTT |  |  |  |  |
|  |  |  |  | Probe¹ | CACAGACCCCGGGACCAACTCAAAATGACC |  |  |  |  |

**Supplemental Table S2. Primer and probe sequences for patient-specific ddPCR**

^1^ Probe on reverse sequence

^2^ Primer concentration 450 nM

^3^ Probe concentration 125 nM
